# Delving into the Therapeutic Potential of *Carica papaya* Leaf against Thrombocytopenia

**DOI:** 10.3390/molecules27092760

**Published:** 2022-04-25

**Authors:** Seemal Munir, Zhi-Wei Liu, Tayyaba Tariq, Roshina Rabail, Przemysław Łukasz Kowalczewski, Jacek Lewandowicz, Andrzej Blecharczyk, Muhammad Abid, Muhammad Inam-Ur-Raheem, Rana Muhammad Aadil

**Affiliations:** 1 National Institute of Food Science and Technology, University of Agriculture, Faisalabad 38000, Pakistan; seemal.munir@gmail.com (S.M.); tayyaba.syed742@gmail.com (T.T.); roshina.rabail@gmail.com (R.R.); 2 College of Food Science and Technology, Hunan Agricultural University, Changsha 410128, China; zwliu@hunau.edu.cn; 3 Department of Food Technology of Plant Origin, Poznań University of Life Sciences, 31 Wojska Polskiego Str., 60-624 Poznań, Poland; 4 Department of Production Management and Logistics, Poznan University of Technology, 2 Jacka Rychlewskiego Str., 60-965 Poznań, Poland; jacek.lewandowicz@put.poznan.pl; 5 Department of Agronomy, Poznań University of Life Sciences, 11 Dojazd Str., 60-632 Poznań, Poland; andrzej.blecharczyk@up.poznan.pl; 6 Institute of Food and Nutritional Sciences, Per Mehr Ali Shah Arid Agriculture University, Rawalpindi 46000, Pakistan; abidfoodscientist@yahoo.com

**Keywords:** thrombocytopenia, *Carica papaya* leaf, phytochemicals, gene expression activity, therapeutic potential

## Abstract

Thrombocytopenia is a clinical manifestation that refers to the low platelet count, i.e., <150 × 10^3^/μL, of blood, resulting in imbalanced hemostasis, which leads to several fatal complications. The causative factors vary greatly, but, as a consequence, they interfere with platelet production and promote destruction, leading to death. *Carica papaya* leaf has unique therapeutic and medicinal characteristics against thrombocytopenia, and this is supported by scientific studies. Secondary metabolites and minerals in the leaf, such as carpaine and quercetin, promote platelet production, inhibit platelet destruction, and maintain platelet membrane through gene expression activity and the ceasing of viral proteases, respectively. This review explores the scientific studies that support the role of papaya leaf in the form of juice, extract, or powder against thrombocytopenia through animal modeling and clinical trials. Phytochemical profiles of *C. papaya* leaf revealed the presence of flavonoids, alkaloids, phenols, cardiac glycosides, tannins, terpenes, and saponins, which impart therapeutic potential to the leaf. The therapeutic benefits of the leaf include immunomodulatory, antiviral, antidiabetic, anticancer, antimalarial, antiangiogenic, antibacterial, and antioxidant activities. Several conducted scientific research studies have proved the efficacy of *C. papaya* leaf against thrombocytopenia, expanding the implication of natural sources to eradicate numerous ailments.

## 1. Introduction

Thrombocytopenia is a hematological condition that is characterized by lower platelets count (< 150 × 10^3^ cells/μL of blood) and is often a multi-factorial disease [1]. Thrombocytes, commonly known as platelets, are blood cells (either nucleated or anucleated) produced in marrow with a normal range of (150–450 × 10^3^) cells/μL of blood [2]. The platelets play a significant role in the physiological system of the body, especially in vascular injury reactions. Platelets forestall the excessive blood loss after injury by forming a structural plug. The various receptors are present in the layers of plug for collagen, ADP, vessel divider von Willebrand factor, and fibrinogen [3]. The platelets have an average life span of 8–10 days, where two-thirds of the platelets are in blood and one-third is in the spleen. The major causes of thrombocytopenia include increased destruction, splenic sequestration, and decreased production of platelets by bone marrow [4]. The causative agents implicate a variety of types of thrombocytopenia, such as drug-induced thrombocytopenia, idiopathic thrombocytopenic purpura, HIV induced, dengue, liver cirrhosis, leukemia, chikungunya, hepatitis C virus, malaria, and various infections [2,5]. The incidence of thrombocytopenia in adults is 3.3/100,000 annually, with a prevalence factor of 9.5 per 100,000 adults [6].

*Carica papaya* is a herbaceous, non-woody, tree-resembling plant that belongs to the *Caricaceae* family [7]. The common name of this evergreen plant is papaya, which has spinally arranged broad leaves (50–70 cm) and bears fruit throughout the year [8]. *C. papaya* is a perennial plant that is native to Southern Mexico and is currently distributed throughout tropical areas worldwide. *C. papaya* fruit has been considered a quasi-drug, and the biological activities of its various parts, including leaves, fruits, shoots, roots, rinds or latex, and seeds, have been proved by several investigations [9]. *C. papaya* leaves have shown multiple therapeutic effects. Its leaves contain high amounts of fat-soluble vitamins (A, D, E, and K); vitamins B and C; and minerals such as iron, sodium, and magnesium [10]. In addition, potassium, nitrogen, and calcium are more absorbed during plant growth, and phosphorus is less extracted [11]. In a few Asian countries, the young *C. papaya* leaves are steamed to eat, similar to spinach, for health benefits. *C. papaya* leaf juice plays a role in liver repair and normalizes clotting by increasing the platelet and white-blood-cell count. Several studies have proved the potential of *C. papaya* leaves in preventing the complications of thrombocytopenia, due to its phytochemical profile [12]. As a result, this review was produced to investigate the most recent studies on *C. papaya* leaves in terms of alleviating thrombocytopenia effects. 

## 2. Mechanism of Action of *C. papaya* against Thrombocytopenia 

*C. papaya* leaf has been scientifically proved to possess therapeutic potential against thrombocytopenia (Table 1). 

**Figure 1:**
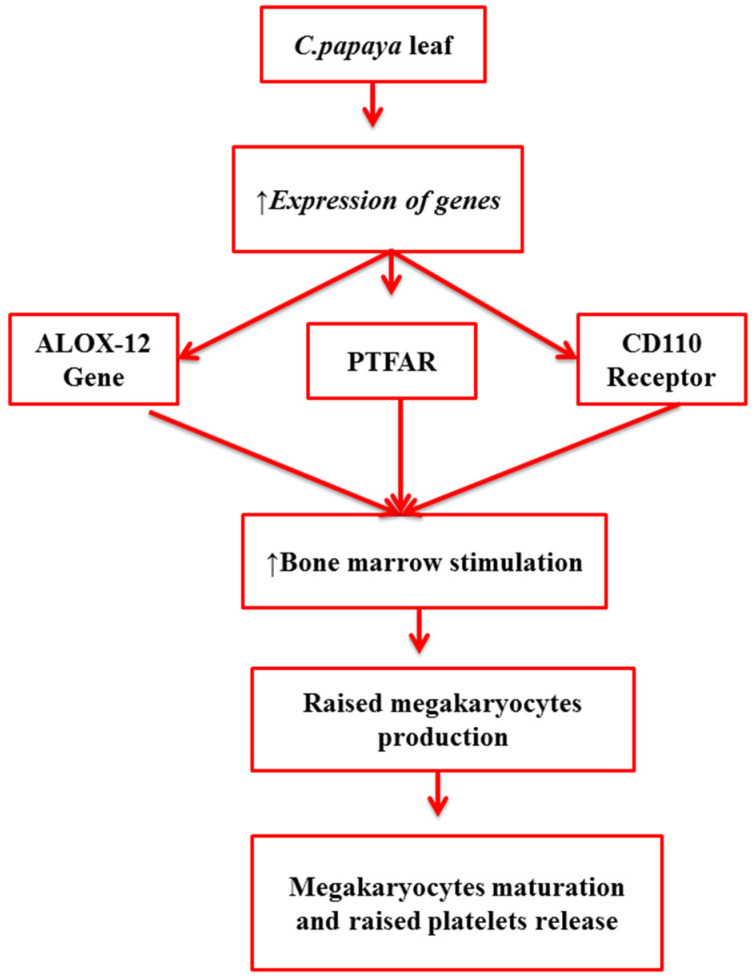
Mechanism of action of *C. papaya* in thrombocytopenia modulation.

It is evident from various studies that *C. papaya* leaf increases platelet production by gene expression activity. The activity of certain genes is increased by the action of *C. papaya* leaf extract, including platelet-activating factor receptor (PTAFR) and arachidonate 12-lipoxygenase (ALOX-12) genes. The high expression of specific genes stimulates the bone marrow to produce more megakaryocytes, as shown in Figure 1. These megakaryocytes are the stem cells responsible for platelet production, and, upon maturation, they break up into small fragments called platelets, hence increasing the platelet production and aggregation in circulating blood [14,16,17].

**Figure 2:**
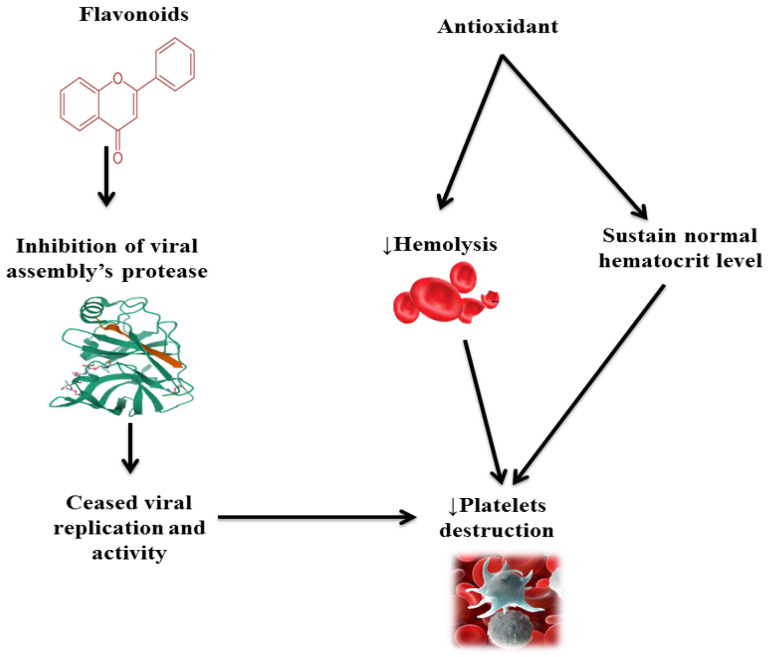
Role of flavonoids and antioxidants of *C. papaya* leaf in inhibiting platelet destruction.

A recent experimental study has shown that carpaine from *C. papaya* leaf is responsible for this mechanism of action [14]. *C. papaya* leaf juice helps to increase the expression of CD110 receptors on the megakaryocytes, which are effective against chemotherapy-induced thrombocytopenia [2]. Thrombocytopenia can also be managed with *C. papaya* leaf by reducing platelets’ destruction. *C. papaya* leaves contain flavonoids that bind to the viral assembly’s proteases, which are the working and replicating units of viruses, to cease viral replication. This process minimizes the platelet destruction and sustains normal hematocrit levels, as elaborated in Figure 2. The leaf extract also exhibits free radical scavenging and antioxidant properties, which impair destruction, thus preventing hemolysis and bleeding. These extracts also enhance the platelet production by increasing the activity of ALOX-12 and PTFAR by 15- and 13–14-folds, respectively [15].

## 3. Selection and Collection of Data

This review is designed to explore available scientific and evidence-based data on *C. papaya* leaf efficacy against thrombocytopenia in various forms, such as juice, extracts, capsules, dried powder, etc., and to interpret their study outcome for the improvement and stability of platelet count. Their ‘therapeutic potential’ and ‘phytochemical screening’ in health promotion and various diseases’ prevention are also evaluated in this review. Therefore, recent accessible experiments on the safe and effective use of the papaya leaf for blood hemostasis and the increase and stability of thrombocyte are collected and studied in line with this scenario. For data collection, an advanced search option on Google Scholar, Science Direct and Scopus has been performed from 2017 to November 2021 with keywords selected as ‘papaya leaf’ OR ‘thrombocytopenia’ AND ‘anti-thrombocytopenic potential’ AND/OR ‘platelet aggregation’. This review commences on the exploration of the anti-thrombocytopenic effects of papaya leaves in relation to drugs, dengue, diseases, etc., and this collective description is not present in the accessed literature yet.

## 4. Thrombocytopenia Ameliorating Potential of *C. papaya* Leaf 

The effectiveness of *C. papaya* leaf extract against thrombocytopenia is inevitable. Recent research has supported its antithrombocytopenia efficacy.

### 4.1. Drug-Induced Thrombocytopenia

The use of drugs has been vigorously increased during the last few decades, including those that affect platelet production and induce platelet destruction, leading to thrombocytopenia. Several drugs have been used for thrombocytopenia induction to determine the mitigating effects of CPL. Bleeding and clotting time were also evaluated, as these parameters are often prolonged during thrombocytopenia. Bleeding time is the time in which bleeding stops after the rupture, while clotting time is the time interval between the start of bleeding and the appearance of fibrin thread. In the following studies, the effect of CPL was investigated on thrombocytopenia, bleeding time, and clotting time. 

#### 4.1.1. Cyclophosphamide-Induced Thrombocytopenia 

Nandini et al. performed an experimental study to evaluate the antithrombocytopenia effect of *C. papaya* leaf. Cyclophosmadide (Cyp) was injected subcutaneously (70 mg/kg BW) for six days to stabilize the thrombocytopenia (210.4 ± 14.2 × 10^3^ cells/μL) in a rat model. Sequential fractionation was used to identify the antithrombocytic phytochemicals in *C. papaya* leaf juice (CPLJ). CPLJ, along with identified fractions, were given to research participants at doses of 200 and 400 mg/kg BD for fourteen days in vivo. The levels of serum thrombopoietin and CD110/cmpl (thrombopoietin receptor on platelets) were measured by using ELISA and Western blotting, respectively. The platelet count was increased to 1073.50 ± 29.6 and 1189.80 ± 36.5 × 10^3^ cells/L, respectively, after CPLJ and butanol fraction administration. Leaf extracts were shown to reduce bleeding time, clotting time, and oxidative indicators. In comparison to the normal and treatment groups, the Cyp-treated group had a slight increase in thrombopoietin (TPO) levels. The expression of the CD110 receptor was found to be low in the Cyp-treated group, but the expression was increased in the CPLJ and butanol fraction treatment groups. The therapeutic efficacy of CPLJ and fractions in mitigating Cyp-induced thrombocytopenia in rat models was discovered by morphological evaluation of megakaryocytes in the bone marrow and histopathology of the liver and kidney [2].

In another study, the efficacy of CPLE on platelet augmentation in Cyp-induced thrombocytopenia rats was investigated. Blood samples from the retro-orbital plexus were taken on the 1st, 4th, 7th, 11th, and 41st days of the study for platelet count evaluation. The importance of CPLE against the envelope and NS1 proteins expression in DENV-infected THP-1 cells was determined by histopathology of the liver, kidney, and spleen on the 14th day and TPO, as well as IL-6 secretion. Erythrocyte damage and hydrogen peroxide–induced lipid peroxidation were reduced, whereas TPO and platelet count were significantly increased [18].

In a Cyp-induced thrombocytopenia animal model, researchers evaluated the effects of standardized aqueous CPLE on platelet count, extramedullary hematopoiesis (EMH), and immunomodulation. For myricetin, caffeic acid, trans-ferulic acid, and kaempferol, the extract was standardized by HPTLC after UPLC–qtof/MS fingerprinting for metabolite signature. The standardized CPLE were provided to Wistar rats (50 and 150 mg/kg) and albino rats (150 mg/kg) for 14 days to assess the effect on platelet and leukocyte proliferation response and immunomodulatory parameters, respectively. The standardized CPLE estimation by quantitative HPTLC exhibited the presence of myricetin, caffeic acid, trans-ferulic acid, and kaempferol, while UPLC–qtof/MS showed the identification of 24 metabolites. Standardized CPLE (150 mg/kg) oral administration exhibited a significant (*p* < 0.01) rise of thrombocytes in the Cyp-induced thrombocytopenia rat group. The results suggest the mediation of CPL in the release of platelets provides means to treat thrombocytopenia [5].

#### 4.1.2. Busulfan-Induced Thrombocytopenia

Busulfan-induced thrombocytopenia rats were investigated to observe the role of thrombopoietin (TPO) in the thrombocytosis effect of CPL. Aqueous and methanol extracts of CPL at 600 mg/kg were administered orally for 7 consecutive days. Intermittent determination of the serum platelet count at the end of the experiment revealed that aqueous and methanol extracts of CPL significantly increased platelet count compared to the control groups (x2 (2) = 25.373, *p* = 0.00). TPO levels were also increased as compared to controls [19]. 

#### 4.1.3. Carboplatin-Induced Thrombocytopenia

In another study, an exploration of *n*-hexane, acetone, ethanol, and methanol extracts of CPLE obtained through Soxhlet apparatus, as well as distilled water extracts prepared by maceration for 8 h, was performed. Carboplatin-induced thrombocytopenia experimental animals were evaluated to observe an increase in platelet count. The findings revealed that thrombocytes rise with no adverse outcomes after hematological and histopathological studies [20].

#### 4.1.4. Cotrimoxazole-Induced Thrombocytopenia

A researcher performed an in vivo study where five groups were formulated by dividing 30 male mice into negative control, positive control, ethanol 96% CPLE dose 0.5 g/kg BW, ethanol 96% CPLE dose 1 g/kg BW, and ethanol 96% CPLE dose 2 g/kg BW. Cotrimoxazole (249.6 mg/kg BW) for 8 days was used to induce thrombocytopenia. The trial results showed the fastest mean time for clotting (*p* < 0.0001), as well as the shortest bleeding time (2.74 ± 0.14), in the group with a CPLE dose of 2 g/kg BW (2.74 ± 0.14) [21].

#### 4.1.5. Hydroxyurea-Induced Thrombocytopenia

Kumar et al. conducted a randomized study on albino rats, which were divided into eight groups (*n* = 6). Hydroxyurea (15 mg/kg) was given orally to induce thrombocytopenia. The first two groups served as saline and toxic control groups. The two different doses of commercial and fresh CPLE were orally given to six treatment groups for five days. The mean platelet count was raised on the 6th day in both low doses (2.06 to 4.93 lakh/mm^3^) and human equivalent dose (2.73 to 7.66 lakh/mm^3^) of commercial extract groups, as compared to the toxic control group (*p* < 0.05). A significant increase in mean platelet count was observed in the group with a human equivalent dose of fresh leaf extract (3.17 to 4.69 lakh/mm^3^), but the low dose of the fresh extract (3.28 to 3.76 lakh/mm^3^) did not show a significant rise. It was concluded that high doses of both fresh and commercial extracts enhance the platelet count, as well as RBC and WBC, with a decrease in bleeding and clotting time [22].

#### 4.1.6. Aspirin-Induced Thrombocytopenia

Antithrombocytopenia efficacy of *C. papaya* fruit and leaf extracts on aspirin-induced thrombocytopenia was observed in male *R. novergicus* albino rats. The fifteen test subjects were separated into three blocks, each containing five rats as the control group, treatment group for fruit extract, and treatment group for leaf extract. The results showed no significant difference in the effect of leaf and fruit, but both enhanced the platelet count, along with the reduction in bleeding and clotting time [23].

#### 4.1.7. Gentamicin-Induced Thrombocytopenia

The subcutaneous subjection of gentamicin (100 mg/kg) for 21 days resulted in an RBC, HGB, and PCV decrease, along with erythrocytic indices alteration, leukocytosis, granulocytosis, and thrombocytopenia. The oral administration of gentamicin and CPL (150 and 300 mg/kg) via a gastric tube for 21 days significantly hindered the drastic effects of gentamicin on the blood profile. The findings also depicted the improvement in erythrogram, leukogram, thrombocytes, EPO, and iron indices in a dose-dependent manner. Thus, CPLE has proved to be an appreciated hemostatic and nephroprotective agent of natural etiology that is good to attenuate blood disorders [24].

### 4.2. Chemotherapy-Induced Thrombocytopenia

Cancer is the leading cause of death globally, and medical science emphasizes the use of chemotherapy to treat this emerging issue. Chemotherapy destroys the cancer cells, as well as the body’s cells, including platelets, thus leading to thrombocytopenia. Several types of research were performed to assess the effectiveness of *C. papaya* leaf in mitigating thrombocytopenia. In 2017, a human-based study was conducted in which forty patients with chemotherapy-induced thrombocytopenia (CIT) were randomly allocated to two groups. The intervention group (*n* = 20) received a 1100 mg dose of CPLE for 7 days after chemotherapy, whereas and no active treatment in the non-intervention group (*n* = 20) was used. The adverse outcomes were assessed by complete hemogram on days 7, 10, 13, 16 and follow up at 28 days after chemotherapy. The intervention group showed a rise in mean platelet count from 49.700 ± 12.649/mm^3^ to 55.350 ± 15.131/mm^3^ (*p* > 0.05), 147.540 ± 54.359/mm^3^ (*p* < 0.01), and 200.585 ± 51.893/mm^3^ (*p* < 0.01) on day 7, 10, 13 and 16, respectively. The non-intervention group showed a mean platelet count of 47.361 ± 13.110/mm^3^, 42.580 ± 12.108/mm^3^, 46.367 ± 14.776/mm^3^, and 54.238 ± 16.053/mm^3^ on respective days. White blood cell increase from baseline to day 7 were statistically significant (*p* < 0.001) as compared to control. Hence, it was concluded that CPLE statistically increased the platelet count by day 13 of post-chemotherapy along with other hematological parameters. Hence, CPLE could be a viable option for the treatment of CIT [25].

Management of CIT by *C. papaya* leaf was observed in another experiment in which the recruitment of thirty subjects as ‘case’ and thirty as ‘control’ was performed. The capsules (290 mg) of CP were given twice a day in ‘cases’ for 5 consecutive days or till normal platelet count. Platelet count increased from 101.93 ± 26.15 × 10^3^/μL to 173.75 ± 29.98 × 10^3^/μL (*p* = 1.37225 × 10^−9^) in “cases” and 99.36 ± 16.62 × 10^3^/μL to 101.75 ± 16.03 × 10^3^/μL (*p* = 0.11) in “controls”. Thus proving that CPL rises platelet count with no adverse effects [26].

A group of scientists after meeting the inclusion and exclusion criteria, assigned 250 patients randomly in a ratio of 1:1 to orally administer thrombobliss (5 mL syrup) twice a day and a placebo for five days. Daily monitoring of platelets showed the platelet elevation from the 2nd day and more evident after 72 h when both mean and median platelet count raised. The cancer patients undergoing chemotherapy elucidated a significantly raised platelet count (*p* < 0.05) by using thrombobliss (CPLE + *Tinospora cordifolia* leaf extract) with no adverse effects [4].

A single-centered retrospective study was directed at an Indian institute where 45 patients were selected with post 50 events of CIT. The malignancy-proven patients were given the standard doses of CPLE to assess therapeutic response. The results suggested that significant platelet count increment occurred in 54% of patients within 5 days, 16% of patients by 7 days, and 18% of patients after more than one week post CPLE administration. The remaining 12% of patients possessed deteriorating platelet counts even after CPLE intervention. Therefore, CIT can be recovered by CPLE therapy in most patients [27].

A prospective study including 40 subjects with CIT who were recruited as case and 20 as control was conducted to assess the responses to UPLAT^®^ and placebo, respectively. UPLAT^®^ (CPLE: 350 mg—2% flavonoids standardization + *Tinospora cardifolia* extract: 150 mg—3% bitters standardization) was given twice a day (2 units each) for 10 consecutive days to cases. The platelet count was significantly higher in the case group (93,990.00 ± 63,896.73) than in the control group (27,600.00 ± 29,758.42), with no side effects associated with treatment in post-chemotherapy cancer patients [28].

### 4.3. Disease-Induced Thrombocytopenia

Several diseases of vital organs, such as decompensated cirrhosis and kidney diseases, also lead to thrombocytopenia. The CPLE and *T. cordifolia* extract potential impact against alcoholic decompensate cirrhosis was investigated by using Cariden (Phytoextracts of CP 1100 mg and *T. cordifolia* 500 mg). The enhancement in platelet count within 15 days was observed by this product, with a normalization period of 90 days in all cases [29].

Another research investigated the preventative impact of CPLE capsules in patients with acute febrile sickness and thrombocytopenia. The patients were randomly assigned to one of two groups (control or intervention) (*n* = 40), with the latter receiving the two capsules thrice a day. The results demonstrated a substantial rise in platelet count and maintenance of normal hematocrit when CPLE capsules were used [30].

Additionally, methanol and acetone aqueous extracts were found to be effective in increasing platelets. The purpose of this study was to determine the extracts’ active involvement in platelet count and bleeding time in rats, while the decocted leaf powder was supplied therapeutically to patients with a low platelet count. Methanol and acetone aqueous extracts and the positive control (heparin) group demonstrated a 68, 56, and 72% reduction in platelet aggregation, respectively. The study found that the *C. papaya* leaf may have the ability to boost platelet count by inhibiting platelet aggregation [31].

Neonates suffering from thrombocytopenia may undergo serious complications, such as intraventricular hemorrhage. Therefore, the CPLE incorporation was performed in the thrombocytopenic neonate. A pre-term newborn with low birth weight was diagnosed with persistent thrombocytopenia and was not responding to any kind of medical treatment. CPLE was administered to the baby thrice a day for 7 days, at a dose of 20 mg/kg. The results showed a normal platelet count without any type of side effects. The well-being of the baby was confirmed by regular follow-ups up to the age of 18 months [15].

In a prospective experimental study, thrombocytopenic children (1–12 years) with dengue hemorrhagic fever grades I and II were included to assess CPLE efficacy. The intervention group consisted of 147 subjects who were given the CPLE syrup with standard therapy, while the control group (*n* = 147) received only the standard therapy for 5 days. The increase in the platelet count was significant in the carpill-treated (CPLE syrup) group in comparison to the control group (*p* < 0.05). The mean-platelet-count increase in the intervention group was 89,739.31, *p* = 0.030, by day 3, and then it was raised to 168,922.75, (*p* = 0.023), by day 5, which is a safe tolerance level [32].

### 4.4. Dengue-Induced Thrombocytopenia

Dengue is a global epidemic which is still a threat to human life. Dengue fever drastically destroys the platelets, leading to thrombocytopenia. In Asian countries, to treat this type of thrombocytopenia, *C. papaya* leaves are used as traditional medicine. In an open study, a total of 500 patients suffering from thrombocytopenia that is associated with dengue fever were divided into study and control groups. CPLE 1100 mg thrice a day for five days consecutively in a week, along with supportive and symptomatic treatment, was provided to the study group, while the control group received only symptomatic and supportive treatment. An increase in the platelet count was observed earlier in the treated group as compared to the controlled group. The average hospitalization time was 5.42 ± 0.98 days and 7.2 ± 0.97 days in the study and control group, respectively [33]. A study was aimed to evaluate the effect of *C. papaya* leaf extract on the count of platelet in patients with dengue fever by including 100 cases. The study group received CPLE 500 mg thrice a day for five days, and at the same frequency, placebo capsules were received by the control group. The study group showed a significant increase (*p* < 0.01) in platelet count compared to the control group; moreover, the study group showed less incidences of complication [16]. CPLE’s role in improving this type of thrombocytopenia was assessed by observing 100 patients divided into two groups. CPLE at 10 mL three times a day was given to the patients, along with kiwi fruit and without any other supportive treatment. The mean platelet count of the study group significantly increased from 212,210 ± 72,257 cells/cumm to 275,282 ± 78,969 cells/cumm (*p*-value = 0.000). The WBC count in the study group also increased compared to the control group after CPLE treatment [34]. Hemorrhagic fever, a complication of dengue, often leads to thrombocytopenia. Another study which had six randomized clinical trials and contained 988 subjects showed an increase in the platelet count in dengue patients after using the *C. papaya* leaf extract. Pooled assessments showed a significant increase in platelet count on day 3 (MD = 12.18; CI 10.28–14.08), day 4 (MD = 31.30; CI 27.77–34.83), and day 5 (MD = 13.23; CI 9.90–16.55). The random-effects model on day 5 also showed a significant increase in platelet count. Furthermore, the studies based on frequency, route of administration, and dosage showed a noteworthy rise in platelet count [35].

A comparative study was carried out in dengue-affected children aged 1 to 16 years with platelets ≤1.5 × 10^5^ per μL and ≥50 × 10^3^/μL. Thirty children were allotted to the study group, as well as to the control group. A 1100 mg tablet prepared with *C. papaya* leaf extract formulated for children above 12 years and syrup at 10 mL for 6-to-12-year-old children and 5 mL for ages below 6 years was given three times a day for 5 days to the study group, and routine symptomatic treatment was given to control group. The platelets count of both groups was monitored and compared. The results indicated that, out of thirty patients, twenty-one were male. The mean platelet count of the study group was 143,270 per μL, while for the control group, it was 148,200 per μL and thus comparable (*p* = 0.76). Meanwhile, on day 5, the mean platelet count was 162,933 per μL and 197,333 per μL in the study and control group, respectively, which showed no significant difference (*p* = 0.15). It was concluded that the extract of *C. papaya* leaf helped increase the platelets in dengue patients as compared to the control group, with no side effects [36]. 

A longitudinal study was conducted including 200 participants who were randomized into two groups. The study group was given CPLE, while the other group had routine supportive treatment. Follow-up from the day of admission to discharge from the hospital revealed a notable difference in mean RBC levels (*p* = 0.045). Moreover, the increase in platelet count in the study group as compared to the placebo group was observed. On the third day, there was a great difference between the study and placebo groups (*p* = 0.002), and the discharge rate was earlier in the test group than in the control group [37].

The platelet-count increase was assessed via the administration of *C. papaya* leaf in dengue patients. Eleven patients were provided with fruits such as *C. papaya*, apples, guava in crumbs, and a spoonful of powdered *C. papaya* leaves with 8 h gape. Patients were also receiving the symptomatic treatment for dengue. A platelet count rise, as well as a decrease in dengue hemorrhagic fever and dengue shock syndrome incidences, was observed [38]. Indian scientists evaluated the efficacy of CPLE and CPLE safety in severe thrombocytopenia management in dengue infection. Adult dengue patients (*n* = 51) with platelet counts 30,000/L were randomly assigned to the treatment group (*n* = 26) or the placebo group (*n* = 24). On day 3, patients treated with CPLE had a significant (*p* = 0.007) increase in platelet counts (482% ± 284) compared to the placebo (331% ± 370) group. Patients in the treatment group required fewer platelet transfusions (1/26 vs. 2/24), and their median time to reach 50,000/L was 2 days (IQR 2–3), compared to 3 days (IQR 2–4) in the placebo group. CPLE was a safe and well-tolerated procedure that did not result in a substantial reduction in mean hospitalization time. Plasma cytokine profiling revealed that the study group faced a lower rise in TNF and IFN levels than the placebo group [39]. Table 2 shows the studies regarding thrombocytopenia ameliorating the potential of *C. papaya*.

## 5. Phytochemical Screening

*C. papaya* L. contains a high concentration of phenolic compounds and minerals, which appear to be responsible for its high antioxidant capacity and may help to prevent and cure ailments [40]. The phytochemical analysis is required to investigate the pharmacological potential of plant extracts and their fractions. The phytochemical screening of fruit and leaves extracts of *C. papaya* reveals the presence of alkaloids, flavonoids, cardiac glycosides, saponins, phenols, tannins, and terpenes. Iskandar and Mustarichie [41] investigated the pharmacognostic parameters of the Indonesian Pharmacopeia of *C. papaya.* The major phytocomponent, 2-ethyl-1-hexanol, in *C. papaya* leaf extract in terms of relative abundance originated from Padang (92.50%), Bandung (76.78%), and Sumedang (32.17%) [41].

The ethanolic leaf extracts of *C. papaya* showed the greatest levels of total phenols (49.24 ± 2.16 mg GAE equivalent/g) and flavonoids (47.16 ± 2.15 mg Rutin equivalent/g) [42]. The most active extracts were ethanolic leaf extracts (25.8 ± 0.5 mm), ethanolic fruit extracts (24.5 ± 0.1 mm), and methanolic leaf extracts (25.8 ± 0.5 mm) (23.4 ± 0.3 mm). The lowest activity was found in the aqueous leaves extract (10.5 ± 0.6 mm). Aqueous extracts showed significantly decreased fungal toxicity when compared to extract prepared using organic solvents. Three dilutions of *C. papaya* leaves and fruit extracts (10, 100, and 1000 g/mL) were made to investigate brine shrimp cytotoxicity. Anticancer components in the form of essential phytonutrients were discovered in the plant extracts. Plant extracts with an LD_50_ of 1000 g/mL were physiologically active, but those with an LD_50_ > 1000 g/mL were not (non-toxic). Similarly, Sarjono et al. [43] reported that the ethanol extract of *C. papaya* leaves contains quinines and steroids. Endophytic bacteria in symbiosis with *C. papaya* leaves produced flavonoids, alkaloid compounds, saponins, triterpenoids, and tannins, all of which exhibit antioxidant properties. Flavonoids such as hesperidin, kaempferol, naringenin, routine, and quercetin were discovered to have the highest antioxidant activity in *C. papaya* leaves. Furthermore, the presence of saponins and alkaloids in complex media binds free radicals. 

Proteolytic enzymes (papain, Caricain chymopapain), alkaloids (carpasemine, carpain), sulfur compounds (benzyl isothiocyanate), triterpenes, flavonoids, oils, and organic acids are all found in *C. papaya* seeds and leaves. *C. papaya* leaf not only contains phenolic compounds, but also contains a considerable amount of saponins. The propensity to donate electrons or hydrogen and generate stable radical intermediates proves phenolic substances can serve as antioxidants. Proteolytic enzymes are an enzymatic group that can hydrolyze the peptide bond in a protein molecule. Papain is a plant proteolytic enzyme (EC 3.4.22.2) that comprises a single polypeptide chain with a sulfhydryl group and three disulfide bridges required for proteolytic activity. Papain has a molecular mass of 23,406 Da and an optimal pH of 6.0–7.0 for maximal activity. Papain is found naturally in all parts of the *C. papaya* plant, making it a very versatile plant that has numerous uses and enzymatic properties [44].

Another study supported the presence of many active components in the leaves of *C. papaya*, including chymopapain, papain, à-tocopherol, cystatin, flavonoids, cyanogenic glucosides, glucosinolates, and ascorbic acid, that have been proven to boost overall antioxidant capacity in blood and lower lipid peroxidation levels [45]. Their active ingredients in *C. papaya* leaves play a role in curing adverse conditions of dengue fever and act as mediators of anti-inflammatory, antitumor, and immunomodulatory activity [14]. Different active compounds of *C. papaya* leaf against certain ailments are listed in Table 3.

## 6. Other Therapeutic Potentials of *C. papaya* Leaf

*C. papaya* leaf has presented a wide range of therapeutic potentials for centuries. The curative roles of the leaf against various ailments include the following: 

### 6.1. Immunomodulatory Activity

Immunomodulation is the process of suppressing the immune system via a particular mechanism. The purpose of this study was to determine the role of freeze-dried *C. papaya* leaf juice (FCPLJ) in avoiding immunological dysregulation in AG129 mice infected with a clinical DENV-2 (DMOF015) isolate. Oral treatment with 500 and 1000 mg/kg/day of FCPLJ for three days was investigated in infected experimental units. Plasma from infected mice included increased levels of GM-CSF, GRO-alpha, IL-1 beta, IL-6, MCP-1, and MIP-1 beta, whereas plasma from uninfected mice contained decreased levels of GM-CSF, GRO-alpha, IL-1 beta, IL-6, MCP-1, and MIP-1 beta. Additionally, there were decreased levels of intracellular IL-6 and viral RNA in the liver. This work established unequivocally that the FCPLJ may have immunomodulatory properties in a non-lethal symptomatic dengue mouse model [51].

The traditional use of *C. papaya* leaf as immune therapy was evaluated in Wistar rats (*n* = 6/group), where 3 doses (0.18, 0.36, and 0.72 mL/100g BD) of mature leaf concentrate were orally administered daily for 3 consecutive days. Ex vivo bone-marrow-cell and splenocyte proliferation, as well as in vitro phagocytic activity of peritoneal macrophages and their accompanying cytokine responses, were assessed by using the mature leaf concentrate of *C. papaya* (MLCC) (31.25, 62.5, 125, 250, 500, and 1000 g/mL). The oral gavage of MLCC increased total leukocytes, platelets, lymphocytes, bone marrow cells, and monocyte subpopulations. Rats’ levels of pro-inflammatory cytokines, Interleukin 6 (IL-6), and Tumor Necrosis Factor (TNF) were drastically decreased by the highest MLCC dosage examined. The oral gavage of all three dosages of the MLCC significantly increased the in vivo phagocytic index of rat PMS. The MLCC improved the phagocytic activity of rat PMS in vitro and induced a Th1-biased cytokine response. Ex vivo proliferation of BMC metha (31.25 g/mL) and SC (31.25 and 62.5 g/mL) was stimulated by MLCC at low doses. In contrast, high quantities (500 and 1000 g/mL) caused cytotoxicity in both BMC and SC, as well as substantial cytokine modulation. The MLCC oral gavage was discovered to have immunomodulatory properties [52].

### 6.2. Antimalarial Activity

In South Asian nations, the usage of *C. papaya* leaf for antimalarial purposes is commonly recognized. In vitro testing was used to assess the effectiveness of *C. papaya* leaf extracts against *Plasmodium falciparum* 3D7 and Dd2 strains. The dichloromethane extract from papaya leaves showed considerable antimalarial efficacy. Bioassay-guided fractionation resulted in a more active carpaine isolate with IC_50_ values of 2.01 ± 0.18 g/mL (4.21 m) and 2.19 ± 0.60 g/mL (4.57 m) against both strains. The leaf extract was shown to be non-toxic to healthy, uninfected human red blood cells, due to its high selectivity. Antimalarial activity was, therefore, demonstrated in this investigation [53]. Swiss mice infected with *Plasmodium berghei* NK65 were given methanolic leaf extract of *C. papaya* to assess if it has antimalarial properties. Six groups of six mice were made from 36 mice in total, with Group A and B acting as normal and disease controls, respectively. Groups C and D received chloroquine (10 mg/kg BW) and artesunate (10 mg/kg BW), whereas Groups E and F received methanolic leaf extract from *C. papaya* at 400 and 600 mg/kg BW, respectively. The *C. papaya* extract decreased parasitemia by 56.76 and 75.53% at 400 and 600 mg/kg BD, respectively, during a curative test, whereas chloroquine and artesunate reduced parasitemia by 92.86 and 90.67% at 10 mg/kg BD, respectively. The extract substantially lowered WBC count and raised HGB and HCT concentration in the treated mice as compared to untreated infected animals. The study discovered that the methanolic leaf extract of *C. papaya* has antimalarial properties [54].

### 6.3. Antiviral Efficacy

Viruses have been causing serious ailments in humans for decades. The pharmaceutical industry has made advancements in antiviral drugs actions and targeting, but these revolutionary amendments have also made the viruses more resistant. The barriers in cure have changed the treatment dimension from synthetic drugs to natural therapeutics. *C. papaya* leaf has exhibited a remarkable role in the pathogenesis of viruses. The effect of freeze-dried CPLJ on viremia and NS1 levels in a dengue fever mice model was potentiated in research [55]. In this investigation, non-mouse adapted New Guinea C strain dengue virus (DEN-2) was inoculated into AG129 mice, followed by oral administration of freeze-dried CPLJ at 500 and 1000 mg/kg/day for three consecutive days.

Twenty-four compounds were identified in freeze-dried CPLJ, and its oral provision decreased the morbidity levels in infected AG129 mice. Another in vitro experimentation was performed for antiviral validation of *C. papaya* leaf on DENV-1 infected C6/36 cells containing plates. The untreated control and four infected/treated samples with *C. papaya* leaf extracts were subjected to the qRT-PCR. The dengue generic real-time RT-PCR (analytical sensitivity of 1 × 10^3^ copies/ M1) was used for DENV-1 RNA quantification by using a negative control, positive control, and four standard dilutions of positive control replicate based on analytical system. The findings revealed less damage in 1/16–1/512 *C. papaya* leaf extract dilutions, according to cell morphology. Thus, 1/32–1/256 dilutions were selected for experimental DENV-1 infection treatment. The qRT-PCR showed DENV RNA in all four standard controls copies, positive control, and DENV-1 infected untreated control, but did not show DENV RNA in the negative control and four DENV-1-treated samples with four different concentrations of papaya leaf extracts. Hence, DENV-1-infection inhibition by treatment with *C. papaya* leaf extract has been validated experimentally [56].

In another recent study, *C. papaya* leaf extract silver–synthesized nanoparticles were subjected to Kidney Vero E2 cell lines for their antiviral evaluation. The aqueous and non-aqueous extractions, followed by silver nanoparticles’ production and characterization, were performed. The possible interactions between the bioactive compounds in *C. papaya* leaf extract and viral NS5 protein were discovered by using molecular docking. Silver nanoparticles were prepared from the methanol extract of *C. papaya* leaves (IC_50_ of 9.20 μg/mL) and showed the most promising action potential. Molecular docking also revealed that 5,7-dimethoxycoumarin is the best ligand for the NS5 protein, with a high affinity. As a consequence, the findings indicated the importance of MCPLE silver nanoparticles in preventing dengue virus type 2 viral replication [7].

### 6.4. Anticancer Properties

*C. papaya* leaf has been proved to fight back various types of cancer, due to its antiproliferative capability. The antiproliferative potential of *C. papaya* leaf juice and its various extracts was investigated on various cell lines representing tumorigenic and normal cells of prostate origin and benign hyperplasia. Molecular-weight-based fractionation revealed antiproliferative responses, growth inhibition, cytotoxic effects, S phase cell cycle arrest, and apoptosis in leaf juice (1–0.1 mg/mL) before and after in vitro digestion (24, 48, and 72 h). The medium polar fraction of leaf juice also prohibited metastatic PC-3 cells from migrating and adhering [57]. The combination of ethanol extracts of Moringa and papaya leaves was administrated in rats as an alternative drug in slowing tumor tissue growth. The combination at a dose of 150 mg/kg BW has been proven to delay the production of cancer tissue [58].

### 6.5. Antidiabetic Activity

Diabetes, in combination with the harsh side effects of synthetic drugs, has encouraged a wider quest for low-cost, low-side-effect alternatives. A study into the antidiabetic effect of ethanolic extract of *C. papaya* leaves was conducted in alloxan-induced diabetic rats. Groups A and B served as positive and negative controls; Group C was given glibenclamide; and Groups D, E, and F were given 200, 400, and 600 mg/kg BD of an ethanolic leaf extract from *C. papaya* leaves, respectively. The oral administration of extracts for 14 days significantly decreased (*p* < 0.05) the blood sugar levels, followed by decreased plasma total cholesterol, triglycerides, and LDL, and increased HDL [49]. In another study, alloxan-induced diabetic mice were used to assess the antidiabetic efficacy of *C. papaya* leaf extract. The following five groups (each with six members) were formed: Groups I and II received 0.1 mL vehicle as a positive and negative control, respectively. Group III received 600 g/kg BD of glibenclamide, while Groups IV and V received 250 and 500 mg/kg of extract. After twenty-eight days of therapy, lipid profiles; blood glucose; and biochemical indicators, such as coronary risk indices and atherogenesis, decreased significantly (*p* < 0.05), while the serum HDL levels increased significantly (*p* < 0.05) [9].

### 6.6. Antiangiogenic Activity

Angiogenesis is the process of forming new blood vessels from existing ones, and it is controlled by a series of on/off switches. The dysregulation of the process may lead to cancer development. The study was carried out by docking the behavior of identified bioactive chemicals present in leaves as ligands with the angiogenic receptors VEGFR1 and VEGFR2, as well as their probable binding sites. Leaf aqueous extract was used to implant the chorioallantoic membrane egg-yolk angiogenesis model. The results showed that *C. papaya* leaf reduced the size, length, and junctions of blood capillaries, as well as the leaf compounds (quercetin, ascorbic acid, lycopene, and riboflavin), and attenuated angiogenesis in pathological conditions [8].

### 6.7. Antioxidative Activity

Oxidative stress tends to be the most critical risk factor, as it results in many life-threatening diseases. The crude extract of *C. papaya* leaves can reduce oxidative stress. An experimental study was monitored in Swiss mice with cyclophosphamide-induced oxidative stress who received 500 mg/kg BD of *C. papaya* leaf extract for 15 days. Studies exhibited that *C. papaya* leaf extract was beneficial against oxidative events, and it prevented DNA damage [50].

The antioxidant and antimutagenic effects of *Anastatica hierochuntica*, *Lepidium sativum*, and *C. papaya* extracts were investigated in Swiss albino mice with Ehrlich ascites carcinoma induced by intraperitoneal injection of EAC-cells. Oral treatment with 500 mg/kg bodyweight of all extracts for 7 days reduced oxidative stress, chromosomal aberration, DNA fragmentation, and inflammation and improved the liver and kidney biomarkers [59].

### 6.8. Antibacterial Activity

Pathogenesis by bacteria has become a major challenge, along with its more resistant responses to treatment drugs. The antibacterial activity of *C. papaya* leaf extracts was observed against *S. aureus*, *B. subtillis*, *E. coli*, and *S. typhi* by using the agar well-diffusion method. The results illustrated the higher activities against tested bacteria with *E. coli* being the maximum. Methanol CPLE fractionation yielded seven fractions, out of which five fractions exhibited the highest activity against *E. coli*. Hence, *C. papaya* leaf extracts can be used in the treatment of urethritis, gastroenteritis, wound infections, and typhoid fever [40]. Table 4 explains the therapeutic potentials of *C. papaya* leaf.

## 7. Conclusions

*C. papaya* is a tropical plant with heavenly potentials in its various parts, especially the leaves. The papaya leaves contain various phytochemicals, such as antioxidants, alkaloids, flavonoids, carpaine, saponins, terpenes, etc. The present review concludes that *C. papaya* leaf has a potent therapeutic effect against various diseases, due to its phytochemical profile. The distinctive characteristics of papaya leaf mitigate diabetes, malaria, viral infections, compromised immunity, oxidative stress, and cancer. A large number of research studies prove that *C. papaya* leaf has an influential role against thrombocytopenia by targeting the ALOX-12, PTFAR, and CD110 receptor genes. The causative agents that induce thrombocytopenia include various drugs, dengue, chemotherapy, and numerous physiological and pathological conditions. The commercially and freshly prepared doses of *C. papaya* leaf equally increase the blood platelet count, as well as impair the platelet destruction without causing any harm. The considerable active antithrombocytopenia components of papaya leaf are phytochemicals that include carpaine, flavonoid, and antioxidants. These components directly influence the respective genes, but further investigations are still required to find out the actual component and its mode of action that alleviate thrombocytopenia.

## Figures and Tables

**Table 1:** Action mechanism of different phytochemicals of *C. papaya* against thrombocytopenia.

Bioactive Component	Mechanism of Action	Reference
Carpaine	An alkaloid Stimulates ALOX-12 and PTFAR genes to produce megakaryocytes that mature and release more platelets Stimulates CD110, i.e., a receptor on megakaryocytes results in high platelet production	[13,14]
Quercetin	A flavonoid Decrease platelet destruction by binding viral proteases Inhibit viral replication that ceases platelet destruction by the viruses	[5]
Antioxidants	Ascorbic acid and beta-carotene Stabilize plate membrane Inhibit hemolysis Decrease platelet destruction	[15]

**Table 2:** Studies showing thrombocytopenia’s ameliorating potential of *C. papaya*.

Thrombocytopenic Settings	Route of Exposure	Methodology	Findings	Reference
Human pediatric subjects aging 1–12 years	Thrombocytopenia associated with DHF grades I and II	*n* = 294, 5 days of treatment Control group (*n* = 147): standard therapy Investigation group (*n* = 147): CPLE syrup + standard therapy	Significant increase in platelets (*p* < 0.05), with good tolerance of extract in peds	[32]
Adult human subjects	Severe thrombocytopenia induced by dengue	*n* = 51, 5 days of treatment Control group (*n* = 24): placebo Investigation group (*n* = 26): CPLE 1100 mg capsule thrice daily	Enhanced platelet aggregation (*p* = 0.023); immunomodulatory and antiviral activity	[39]
Rat model	Cyclophosphamide-induced (6 days of subcutaneous injections of dose 70 mg/kg BD) thrombocytopenia	*n* = 88 (11 groups, 8 animals each), 14 days study Normal; thrombocytopenia and control; Hydrocortisone; CPJ1; CPJ2; CPJ-BT1; CPJ-BT2; CPJ-EA1; CPJ-EA2; CPJ-PE1; CPJ-PE2 (respective doses of 200 and 400 mg/kg bw in study groups)	Enhanced platelet count, reduced bleeding and clotting time, decreased oxidative markers, and increased serum thrombopoietin	[2]
Male Sprague Dawley rats	Cyclophosphamide-induced (s/c injections 50 mg/kg BW for 2 consecutive days) thrombocytopenia	14 days of study, CPLE administration Hematology and histopathology evaluation	Significant platelets reduced NS1 and enveloped protein in DENV-infected THP-1 cells, and increased TPO levels	[18]
Human subjects aged 18 years	Chemotherapy-induced thrombocytopenia	*n* = 40, 7 days of treatment, 28-day follow-up Control group (*n* = 20): placebo Investigation group (*n* = 20): 1100 mg CPLE	Enhanced platelet count (*p* < 0.001), with improved hematological parameters	[25]
Male mice	Cotrimoxazole-induced (249.6 mg/kg BW for 8 days) thrombocytopenia	*n* = 30, 5 groups Groups 1 and 2: negative and positive control group Group 3: 0.5g CPLE/kg BW Group 4: 1 g/kg CPLE Group 5: 2 g/kg CPLE	2 g CPLE/kg BW showed the highest significance, fast bleeding time (*p* < 0.0001)	[21]
Children aged 1 to 16 years	Dengue-associated thrombocytopenia	*n* = 30, 5 days dosage Control group: placebo Investigation group: Above 12 (1100 mg tablet thrice), 6–12 years (10 mL syrup thrice), below 6 (5 mL thrice)	Increasing trends in platelet count, with fast recovery from dengue (*p* = 0.15)	[36]
Humans	Dengue-associated thrombocytopenia	*n* = 988, 6 randomized trials, 5 days of CPLE administration	Platelets count increased; fast recovery from dengue	[35]
Humans	Dengue-associated thrombocytopenia	*n* = 100, 3 days dosage Control group (*n* = 50): Placebo Test group (*n* = 50): adult (CPLE 10 mL thrice daily + 1 kiwi fruit) Children (CPLE 5 mL thrice + 1 kiwi fruit)	Increased platelets and WBC count (*p* = 0.000), reduced muscle pain, and skin rashes	[34]
Infants	Neonatal thrombocytopenia	Neonate with sepsis, 7 of days treatment, follow-ups up to 18 months, 20 mg CPLE thrice daily	Platelet’s production increased with no side effects or discomfort	[15]
Humans	Chemotherapy-induced thrombocytopenia	*n* = 60, 5 days of study Control group (*n* = 30): placebo Case group (*n* = 30): CPLE capsules (290 mg twice daily)	CP leaf extracts significantly increased thrombocytes in post-chemotherapy cancer patients (*p* = 0.11) No adverse effects	[26]
Male patients	Alcoholic decompensate liver-cirrhosis-induced thrombocytopenia	*n* = 3 Cariden (phytoextracts of CP—1100 mg + *Tinospora cordifolia*—500 mg)	Enhances platelets in 15 days and normalizes level in 90 days of therapy	[29]
Humans	Chemotherapy- and microbial-infection-induced thrombocytopenia	*n* = 250, 5 days randomized study Control group (*n* = 125): placebo Intervention group (*n* = 125): 5 mL syrup twice daily	*C. papaya* and *T. cordifolia* leaves extracts combination effectively increases platelet count (*p* < 0.05)	[4]
Male albino rats	Nephrotoxicity due to subcutaneous administration of gentamicin	21 days CPLE administration (150 and 300 mg/kg BW orally) Kidney biomarkers and blood profiling performed	Exhibit nephroprotective effect; increased RBCs, platelet, WBCs, HGB, and iron-binding capacity	[24]
Sprague Dawley rats	Intraperitoneal injection of carboplatin	*n* = 48, 7 groups (*n* = 6 each) 21 days dosage Control group: 0.3 mL distilled water Investigation group: 400 mg/kg BW	Raised platelets’ levels significantly	[20]
Humans	Chemotherapy-induced thrombocytopenia	*n* = 45, treatment period varied (7–14 days) 1100 mg tablets twice daily till recovery	Improved platelet count, mitigation of treatment delay in CIT	[27]
Female albino Wistar rats	Cyclophosphamide-induced thrombocytopenia	*n* = 24, 4 groups (*n* = 6 each group), 14 days treatment Groups I and II: normal and toxic control—0.8 mL saline water Group III: 50 mg/kg BW SCPLE Group IV: 150 mg/kg BW SCPLE	↑ Platelet, leukocyte count in treatment groups (*p* < 0.01) ↓ Bleeding and clotting time in Groups III and IV Immunomodulation in SCPLE treated groups Highest significance in Group IV	[5]
Humans	Dengue-associated thrombocytopenia	*n* = 100, 5 days treatment Study group (*n* = 50): CPLE capsules at 500 mg thrice daily Control group (*n* = 50): placebo capsules thrice daily	↑ Platelet count significantly in the study group (*p* < 0.01) ↓ Thrombocytopenia complications	[16]
Humans	Chemotherapy-induced thrombocytopenia	*n* = 60, 10 days of treatment Control group (*n* = 20): placebo Case group (*n* = 40): UPLAT^®^ twice daily	Cases group showed an increased thrombocyte count in post-chemotherapy, no adverse effects	[28]
Preclinical: Wistar albino rats Clinical: Humans	Dengue-associated thrombocytopenia	Preclinical: 400 mg/kg BW of ME CPLE and AC CPLE and heparin 100 mg/kg BW Clinical: 1g leaf powder thrice daily	Pre-clinical: faster bleeding time by 3, 4, and 7 s respectively Clinical: platelet count increased by platelet attenuation	[31]
Sprague Dawley rats	Busulfan-induced thrombocytopenia (20 mg/kg BW intraperitoneal injections for 3 days)	*n* = 32, 7 days treatment, 4 groups (*n* = 8) Group A and B: positive and negative control Group C: 2 mL/day AQ CPLE Group D: 2 mL/day ME CPLE	Increased platelets (*p* = 0.00) and TPO (*p* = 0.149) levels in treatment groups, AQ CPLE showed the best results	[19]
Humans	Dengue-associated thrombocytopenia	*n* = 80, Control group (*n* = 40): placebo Investigation group (*n* = 40): 2 CPLE capsules thrice daily	Significant rise in platelet count (*p* < 0.05) Normal hematocrit level stability	[30]
Humans	Dengue-induced thrombocytopenia	*n* = 500, 5 days of dosage Study group: 1100 mg thrice daily	The study group showed a clear increase in Thrombocytes, and reduced dengue complications	[33]
Humans	Dengue-induced thrombocytopenia	*n* = 200 Control group (*n* = 100): standard treatment Investigation group (*n* = 100): CPLE	Increased platelet count (*p* = 0.045), reduced hospitalization, used as a supplement	[37]
Albino rats	Hydroxyurea (15 mg/kg BW orally)	*n* = 48, 5 days dosage, eight groups (*n* = 6) Group 1 and 2: control and toxic control The remaining 6 groups were fed with different doses of commercial and fresh CPLE	Significant increase in mean platelet, RBCs, and WBCs (*p* < 0.05) Decreased bleeding and clotting time	[22]
Male *Rattus novergicus* albino rats	Aspirin-induced thrombocytopenia	*n* = 15, 3 groups (*n* = 5), Group 1: control Group 2: CPFE Group 3: CPLE	Leaf extract showed a more significant increase in platelet, reduced bleeding and clotting time	[23]
Human	Dengue thrombocytopenia	*n* = 1 *C. papaya* + guava + apple + 1 spoonful ground leaf every 8 h	Increased platelets, reduced incidence of dengue	[38]

**Table 3:** Active compounds of *C. papaya* leaf against different ailments.

Activity	Bioactive Component	Mechanism of Action	Reference
Immunomodulation	Rutin and Narirutin	Stimulate JNK and ERK pathways in macrophages Increase prostaglandins 2 and pro-inflammatory cytokines Act as immunity-enhancing supplement	[46]
Antimalarial	Papain and Chymopapain	Cause immunity against insect attack Show larvicidal activity against *Anopheles stephensi* Combat malaria	[47]
Antiviral	Quercetin	Binds viral proteases Ceases viral replication Inhibits and destroy viral activity	[7]
Anticancer	Flavonoids	Cause cell-cycle arrest in cancer cells Promote apoptosis of cancer cells Inhibit reactive oxygen species Prevent cancer	[48]
Anticancer	Flavonoids	Cause cell-cycle arrest in cancer cells Promote apoptosis of cancer cells Inhibit reactive oxygen species Prevent cancer	[49]
Antidiabetic	Polyphenols	Hypoglycemic effect Inhibition of carbohydrate-hydrolyzing enzyme, α-amylase, and α-glucosidase Lower and retard glucose absorption	[45]
Antiangiogenic	Lycopene and Quercetin	Reduce length, size, and junction of blood vessels Inhibit the formation of abnormal new blood vessels Combat angiogenesis	[8]
Antioxidation	Ascorbic acid and tocopherol	Destroy ROS activity Inhibit oxidation of cell integrity Restraining oxidative chain reaction	[50]
Antibacterial	Alkaloids	Inhibit bacterial growth Prevent bacterial colonies formation Destroy bacteria	[40]
Immunomodulation	Rutin and Narirutin	Stimulate JNK and ERK pathways in macrophages Increase prostaglandins 2 and pro-inflammatory cytokines Act as immunity enhancing supplement	[46]
Antimalarial	Papain and Chymopapain	Cause immunity against insect attack Show larvicidal activity against Anopheles stephensi Combat malaria	[47]
Antiviral	Quercetin	Binds viral proteases Ceases viral replication Inhibits and destroy viral activity	[7]
Antidiabetic	Polyphenols	Hypoglycemic effect Inhibition of carbohydrate-hydrolyzing enzyme, α-amylase, and α-glucosidase Lower and retard glucose absorption	[49]
Antiangiogenic	Lycopene and Quercetin	Reduce length, size, and function of blood vessels Inhibit the formation of abnormal new blood vessels Combat angiogenesis	[8]
Antioxidation	Ascorbic acid and tocopherol	Destroy ROS activity Inhibit oxidation of cell integrity Restrain oxidative chain reaction	[50]
Antibacterial	Alkaloids	Inhibit bacterial growth Prevent bacterial colonies formation Destroy bacteria	[40]

**Table 4:** Therapeutic potentials of *C. papaya* leaf.

Activity	Specimen	Route of Exposure	Methods	Findings	Reference
Immunomodulation	AG 129 Mice	Clinical DENV-2 (DMOF015) Isolate infection	2-phase study, *n* = 25, 5 groups, 3 days post-infection dosing of 500 and 1000 mg/kg BW of freeze-dried CPLJ	WBC and neutrophils augmentation, anti-inflammatory activity, adjunctive immunotherapy	[51]
	Wistar rats	No disease induction	*n* = 24. 4 groups, 3 days dosing of distilled water, 0.18, 0.36, and 0.72 mL mature leaf concentrate/100g of BW	Increased blood cells, improved functional and non-functional immune parameters	[52]
Antimalarial	In vitro, leukocyte-depleted RBC	*P. falciparum* 3D7 and Dd2 strains	Bioassays/fractionation, carpaine isolation from leaf extraction, cytotoxicity evaluation against NL20 cells, and hemolysis assay performance on carpaine	Carpaine showed good activity against both strains, selective against the parasite, non-toxic to healthy RBCs, cured malaria	[53]
	Swiss Mice	Intraperitoneal inoculation of *P. berghei NK65* strain	*n* = 36, 6 groups, alternative dosages of standard drugs (10 mg/kg BW) and methanol CPLE (400 and 600 mg/kg BW)	Methanol CPLE act as an antimalarial entity, reduced WBC, increased HGB and HCT	[54]
Antiviral	AG 129 Mice	Non-mouse adapted New Guinea C Strain Dengue virus inoculation	*n* = 20, 4 groups, 3-day post-infection dosing of mock doses, 500 and 1000 mg/kg BW of freeze-dried CPLJ	Decreased morbidity levels in the infected specimen by FCPLJ	[55]
	C6/36 Cell containing culture	DENV-1 infected cells	Controls and 4 treated with four different dilutions of CPLE, qRT-PCR performed	Inhibition of DENV-1 viral infection in all four samples	[56]
	Kidney Vero E2 Cell lines	DENV-2 NS5 protein	Molecular docking, methanol CPLE Silver synthesized nanoparticles evaluation against dengue	Anti-dengue efficacy of silver synthesized nanoparticles of CPLE was observed in vitro	[7]
Anticancer	Cell lines of prostate origin	Cell proliferation assays	In vitro, time-course analysis, CPL juice (1–0.01 mg/mL) with various extracts validated against the range of proliferative cell lines	Antiproliferative response and antimetastatic potential	[57]
	Rats (breast gland)	1 mL/rat DMBA induction with a dose of 25 mg	Combination of ethanolic extracts of moringa and *C. papaya* leaves, 150 and 200 mg/kg BW dosing	150 mg/kg BW slowed cancer and tumor growth	[58]
Antidiabetic	Albino rats	Intraperitoneal Alloxan monohydrate injection (150 mg/kg BW)	*n* = 30, 5 groups of distilled water, drugs, and ethanolic CPLE (250 and 500 mg/kg BW), 28 days dosing	Antihyperglycemic efficacy and improved lipid profiles	[9]
	Albino rats	Alloxan monohydrate + sterile saline induced (150 mg/kg BW)	*n* = 30, 6 groups, 14 days dosing with DW, drugs, and ethanolic CPLE (200, 400, and 600 mg/kg BW)	Decreased blood glucose, hypolipidemia, lower TC, LDL, HDL, and TG	[49]
Antiangiogenic	Fertilized chicken eggs	No induction	Humidified incubation, small window cut, control group, and various aqueous CPLE treated groups, 3-day incubation	Attenuate angiogenesis, reduction in length, size, and the junction of blood vessels	[8]
Antioxidant	Male Swiss Mice	Cyclophosphamide induced (75 mg/kg BW)	15 days treatment, groups received water, drug, and CPLE (500 mg/kg BW)	Exhibit potential against oxidative events, reduce free radicals, and prevent DNA damage	[50]
	Female Swiss albino mice	Intraperitoneal injections of Ehrlich ascites carcinoma cells	*n* = 90, 9 groups, provided with placebo, drugs, and 500 mg/kg BW of AH, LP, and CP extracts	Antioxidant, antimutagenic, and antitumor activity	[59]
Antibacterial	In vitro, Petri-dish culture	*Staphylococcus aureus*, *Bacillus subtilis*, *Salmonella typhi*, and *Escherichia coli* inoculation	Positive and negative control and methanolic CPLE treated groups validation against various bacterial strains, MCPLE 7 component fractionation	Antibacterial activity, highest against *E. coli* by 5 components of MCPLE, treat gastroenteritis, urethritis, and wound infections	[40]

## Data Availability

Not applicable.

## Abbreviations

BMC	Bone marrow cells
BW	Body weight
CP	*Carica papaya*
CPLE	*Carica papaya* leaf extract
CPLJ	*Carica papaya* leaf juice
CIT	Chemotherapy-induced thrombocytopenia
Cyp	Cyclophosphamide
DENV	Dengue virus
DHF	Dengue hemorrhagic fever
EPO	Erythropoietin
FCPLJ	Freeze-dried *Carica papaya* leaf juice
GM-CSF	Granulocyte-macrophage colony-stimulating factor
GRO-alpha	Growth regulated oncogene
HDL	High-density lipoprotein
HGB	Hemoglobin
HIV	Human immunodeficiency virus
HPTLC	High-performance thin-layer chromatography
IC_50_	Inhibiting concentration
IL	Interleukin
LDL	Low-density lipoprotein
MCP-1	Monocyte chemoattractant protein
MIP-1	Macrophage inflammatory protein
MLCC	Mature leaf concentrate of *Carica papaya*
mm	Millimolar
NS	Non-structural protein
PCV	Packed cell volume
qRT-PCR	Real-time polymerase chain reaction
RBCl	Red blood cells
RNA	Ribonucleic acid
SC	Stem cells
SCPLE	Standardized *Carica papaya* leaf extract
TPO	Thrombopoietin
UPLC–qtof/MS	Ultra-performance liquid chromatography–quadrupole time of flight/mass spectrometer
VEGFR	View/edit human vascular endothelial growth factor receptor
WBC	White blood cells
